# An Efficient Synthesis of Spiro[indoline-3,9′-xanthene]trione Derivatives Catalyzed by Magnesium Perchlorate

**DOI:** 10.3390/molecules22081295

**Published:** 2017-08-04

**Authors:** Chunfeng Chen, Chunlei Lv, Jianfeng Liang, Jianqing Jin, Lijun Wang, Chunlei Wu, Runpu Shen

**Affiliations:** 1 Department of Chemistry and Chemical Engineering, Shaoxing University, Shaoxing 312000, Zhejiang, China; ljwang@usx.edu.cn; 2 Zhejiang Medicine Co., Ltd., Shaoxing 312000, Zhejiang, China; chenchunfeng@zmc-china.com (C.C.); lcl@zmc-china.com (C.L.); ljf@zmc-china.com (J.L.); jjq@zmc-china.com (J.J.)

**Keywords:** aqueous, isatin, spiro[indoline-3,9′-xanthene]trione, magnesium perchlorate

## Abstract

A simple and efficient method for the synthesis of spiro[indoline-3,9′-xanthene]trione derivatives by means of condensation between isatins and 1,3-cyclohexanedione in the presence of a catalytic amount of magnesium perchlorate at 80 °C in 50% aqueous ethanol medium has been described. Notably, the present method offers desirable advantages of good yields, simplicity of workup procedure, easy purification, and reduced reaction times.

## 1. Introduction

Heterocyclic compounds play important roles in the drug discovery process. The indole moiety is probably the most well-known heterocycle, which occurs in many important natural products, pharmaceuticals, and other synthetic materials exhibiting a variety of biological activities and other properties [1,2]. Spiro compounds represent an important class of naturally occurring substances characterized by highly pronounced biological properties [3]. The spirooxindole system is the core structure of many pharmacological agents and natural alkaloids [4,5,6]. The key structural characteristic of these compounds is the spiro ring fused at the 3-carbon position of the oxindole core with varied heterocyclic motifs. These spirooxindoles seem to be promising candidates for drug discovery, since they incorporate both oxindoles and other heterocyclic moieties simultaneously. For example, MI-219, a spiroindoline-3,3′-pyrrolidine, was described by Wang et al. as a potent MDM2 inhibitor [7]. Xanthenes have been reported to possess pharmacological activities such as antiviral, antibacterial, and anti-inflammatory properties, and are also known as sensitizers in photodynamic therapy (PDT) for abolishing tumor cells [8,9,10,11].Spiro[indoline-3,9′-xanthene]trione, as a member of spiro compounds, has attracted researchers’ attentions for its unique structure and biological effects since the first report by Lorenc in 1959 [12], and various catalysts for its synthesis have been developed such as *p*-*tert*-Butylcalix[8]arene [13], *p*-TSA [14,15], ZnO [16], ZnFe_2_O_4_ [17], B(HSO_4_)_3_ [18], HBF_4_ supported on silica nanoparticles [19], and SnCl_4_ [20]. Although these reported protocols have their eligibilities, they also suffer from one or more drawbacks. So, the development of novel, efficient methods is still a valid goal.

In recent years, magnesium perchlorate has received considerable attention as an inexpensive, nontoxic, readily available catalyst for various transformations under mild and convenient conditions, affording the corresponding products in excellent yields with high selectivity [21,22,23,24,25]. Our previous work on the catalytic synthesis of spiro[4*H*-pyran-oxindole] derivatives was carried out smoothly in the presence of magnesium perchlorate [26]. The handling, storage, and precautionary information of the oxidative Mg(ClO_4_)_2_ can be found in the literature, and it is relatively safe when used as a Lewis acid catalyst [27,28].

**Scheme 1:**
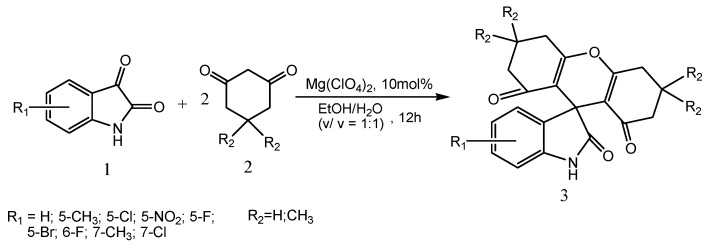
Synthesis of spiro[indoline-3,9′-xanthene]trione.

In continuance of our research on the synthesis of spiro compounds and the application of perchlorate, we herein investigated the synthesis of a series of spiro[indoline-3,9′-xanthene]trione via the condensation of isatin and 1,3-cyclohexanedione in the presence of green catalyst magnesium perchlorate in aqueous ethanol medium (Scheme 1).

## 2. Results and Discussion

In our initial study, the reaction of isatin **1a** and dimedone **2** was used as a simple model substrate in different solvents in the presence of magnesium perchlorate to achieve suitable conditions for the synthesis of spiro[indoline-3,9′-xanthene]trione. After screening, we found that the reaction can proceed smoothly in good yield in aqueous ethanol solution (50%, *v*/*v*). The reaction mixture was presented as a suspension state throughout all the process. The color of the reaction mixture changed from pale red to pale yellow along with the reaction proceeding. The results are summarized in Table 1.

As shown in Table 2, it was found that this procedure works with a wide variety of substrates. Nine types of substituted isatins as well as 1,3-cyclohexanedione were used in this reaction (Scheme 1). After the reaction was over monitored by the thin layer chromatography (TLC), the resulting solid was filtered and washed with aqueous ethanol solution to yield a crude product, which was then recrystallized from ethanol to afford pure substituted spiro[indoline-3,9′-xanthene]trione **3a**–**3m**. All of the products were crystalline and characterized based on their melting points, elemental analysis, and spectral data (IR, ^1^H-NMR, ^13^C-NMR).

**Figure 1:**
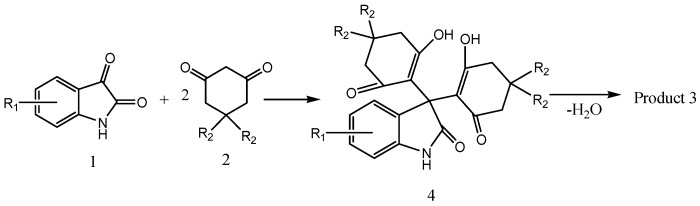
Proposed mechanism for the synthesis of spiro[indoline-3,9′-xanthene]trione.

The mechanism for the synthesis of spiro[indoline-3,9′-xanthene]trione was via the intermediate **4**, followed by the removal of a molecule of water (Figure 1), which can be proved by the following process. 6-Fuloro-isatine and dimedone were stirred for 24 h at room temperature and then catalyzed by magnesium perchlorate in the aqueous ethanol. Then the precipitate was filtered, washed and dried. The ^1^H-NMR spectra data of the obtained solid showed that there was a strong peak in the shift of 11.68, which was assigned to the hydroxyl hydrogen of the compound **4**. Finally, the solid was stirred at 80 °C for another 10 h to afford the product **3m**. This proposed mechanism is in consistent with that reported in References [15,29,30].

## 3. Experimental Section

All chemicals used were obtained from commercial suppliers and used without further purifications. IR spectra were recorded on a Nicolet-6700 spectrometer (Thermo Scientific, MMAS, Waltham, MA, USA). ^1^H-NMR spectra were determined on a Bruker AVANCE DMX III 400 M spectrometer (Bruker, Fallanden, Switzerland) and ^13^C-NMR spectra were obtained on the same instrument. Samples were dissolved in deuterated DMSO, which provided the deuterium lock for the spectrometers. Elemental microanalysis was carried out on a Euro vector EA 3000 CHN analyzer (Milan, Italy). Melting points were measured using a BUCHI M-560 melting point apparatus (Buchi labortechnik AG, Flawil, Switzerland). HRMS measurements were performed on a Waters Micromass GCT Premier (Waters, MMAS, New York, NY, USA). Reactions were monitored by thin-layer chromatography (TLC), carried out on 0.25 mm silica gel plates and visualized with UV light.

### General Procedure for the Synthesis of Spiro[indoline-3,9′-xanthene]trione *(**3**)*

Mg(ClO_4_)_2_ (0.1 g) was added to a mixture of isatin (2 mmol), and dimedone (4 mmol) in aqueous ethanol solution (50%, *v*/*v*, 5 mL), and the resulting mixture was stirred at 80 °C for 10–12 h. Upon completion of the reaction (TLC, Ethyl acetate/Petroleum ether = 1:4), the mixture was allowed to cool to room temperature. The resulting solid was filtered and washed successively with water (2 × 30 mL) and cold aqueous ethanol (2 × 1 mL) to afford a crude product, which was recrystallized from EtOH to afford the pure product **3**. Some insolubility could be observed in the refluxing ethanol during the recrystallization process, which should be filtered when hot.

*3′,3′,6′,6′-Tetramethyl-3′,4′,6′,7′-tetrahydrospiro[indoline-3,9′-xanthene]-1′,2,8′(2′H,5′H)-trione* (**3a**). White powder; m.p.: 306–307 °C (305 °C) [15]; IR (KBr) cm^−1^: 3431, 2957, 1733, 1666, 1616, 1469, 1345, 1222, 1168. ^1^H-NMR (400 MHz, CDCl_3_): δ_H_ (ppm) 1.04 (6H, s, 2CH_3_), 1.12 (6H, s, 2CH_3_), 2.11 and 2.25 (4H, AB system, *J* = 16 Hz, 2CH_2_), 2.44 and 2.56 (4H, *J* = 17.6Hz, 2CH_2_), 6.83–6.90 (3H, m, H-Ar), 7.13–7.17 (1H, m, H-Ar), 7.62 (1H, s, NH); ^13^C-NMR (100 MHz, CDCl_3_): δ_C_ (ppm) 27.2, 29.0, 32.0, 41.2, 45.7, 50.9, 109.4, 113.6, 121.9, 122.3, 128.5, 133.6, 142.3, 163.5, 178.8, 195.3; Anal. Calcd. for C_24_H_25_NO_4_: C, 73.64; H, 6.44; N, 3.58%. Found: C, 73.67; H, 6.36; N, 3.52%.

*5,3′,3′,6′,6′-Pentamethyl-3′,4′,6′,7′-tetrahydrospiro[indoline-3,9′-xanthene]-1′,2,8′(2′H,5′H)-trione* (**3b**). White powder; m.p.: 294–295 °C (310 °C) [16]; IR (KBr) cm^−1^: 3314, 2959, 1745, 1670, 1618, 1349, 1315, 1168. ^1^H-NMR (CDCl_3_): δ_H_ (ppm) 1.04 (6H, s, 2CH_3_), 1.12 (6H, s, 2CH_3_), 2.13 and 2.24 (4H, AB system, *J* = 16.4 Hz, 2CH_2_), 2.21 (3H, s, CH_3_), 2.45 and 2.55 (4H, AB system, *J* = 17.6 Hz, 2CH_2_), 6.66 (1H, s, H-Ar), 6.73 (1H, d, *J* = 8.0 Hz, H-Ar), 6.95 (1H, d, *J* = 7.6 Hz, H-Ar), 7.46 (1H, s, NH). ^13^C-NMR (CDCl_3_): δ_C_ (ppm) 21.2, 27.4, 28.9, 31.9, 41.2, 45.7, 50.9, 108.9, 113.7, 123.2, 128.9, 132.2, 133.7, 139.8, 163.4, 178.7, 195.3. Anal. Calcd. for C_25_H_27_NO_4_: C, 74.05; H, 6.71; N, 3.45%. Found: C, 73.58; H, 6.41; N, 3.48%.

*5-Chloro-3′,3′,6′,6′-tetramethyl-3′,4′,6′,7′-tetrahydrospiro[indoline-3,9′-xanthene]-1′,2,8′(2′H,5′H)-trione* (**3c**). White powder; m.p.: 309–310 °C; IR (KBr) cm^−1^: 3457, 2958, 1739, 1665, 1616, 1509, 1349, 1224, 1120. ^1^H-NMR (CDCl_3_): δ_H_ (ppm) 1.06 (6H, s, 2CH_3_), 1.12 (6H, s, 2CH_3_), 2.16 and 2.26 (4H, AB system, *J* = 16.4 Hz, 2CH_2_), 2.48 and 2.56 (4H, *J* = 17.6 Hz, 2CH_2_), 6.76 (1H, d, *J* = 8.4 Hz, H-Ar), 6.83 (1H, d, *J* = 1.6 Hz, H-Ar), 7.12 (1H, dd, *J* = 1.6 Hz, *J* = 8.4 Hz, H-Ar), 7.89 (1H, s, NH). ^13^C-NMR (CDCl_3_): δ_C_ (ppm) 27.5, 28.7, 31.9, 41.2, 45.8, 50.9, 110.3, 113.2, 122.6, 126.9, 128.3, 135.2, 141.2, 163.8, 178.3, 195.4. Anal. Calcd. for C_24_H_24_ClNO_4_: C, 67.68; H, 5.68; N, 3.29%. Found: C, 67.59; H, 5.62; N, 3.37%. HRMS (ESI): *m*/*z* [M + H]^+^ calcd. for C_24_H_24_ClNO_4_: 425.9047; found: 425.9041.

*5-Nitro-3′,3′,6′,6′-tetramethyl-3′,4′,6′,7′-tetrahydrospiro[indoline-3,9′-xanthene]-1′,2,8′(2′H,5′H)-trione* (**3d**). Pale yellow powder; m.p.: 276–277 °C (278 °C) [15]; IR (KBr) cm^−1^: 3452, 2958, 1745, 1667, 1629, 1346, 1225, 1169. ^1^H-NMR (CDCl_3_): δ_H_ (ppm) 1.00 (6H, s, 2CH_3_), 1.11 (6H, s, 2CH_3_), 2.17 and 2.26 (4H, AB system, *J* = 16 Hz, 2CH_2_), 2.53 and 2.59 (4H, AB system, *J* = 18 Hz, 2CH_2_), 6.89 (1H, d, *J* = 8.8 Hz, H-Ar), 7.80 (1H, d, *J* = 2.0 Hz, H-Ar), 8.14 (1H, dd, *J* = 2.0 Hz, *J* = 8.8 Hz, H-Ar), 8.37 (1H, s, NH). ^13^C-NMR (CDCl_3_): δ_C_ (ppm) 27.7, 28.5, 32.1, 41.1, 45.5, 50.8, 108.9, 112.8, 118.1, 125.9, 134.5, 143.0, 148.6, 164.5, 178.6, 195.8. Anal. Calcd. for C_24_H_24_N_2_O_6_: C, 66.04; H, 5.54; N, 6.42%. Found: C, 65.91; H, 5.47; N, 6.29%.

*7,3′,3′,6′,6′-Pentamethyl-3′,4′,6′,7′-tetrahydrospiro[indoline-3,9′-xanthene]-1′,2,8′(2′H,5′H)-trione* (**3e**). White powder; m.p.: 287–289 °C; IR (KBr) cm^−1^: 3336, 2955, 1731, 1671, 1618, 1348, 1224, 1168. ^1^H-NMR (CDCl_3_): δ_H_ (ppm) 1.04 (6H, s, 2CH_3_), 1.12 (6H, s, 2CH_3_), 2.15 and 2.25 (4H, AB system, *J* = 17.2 Hz, 2CH_2_), 2.27 (3H, s, CH_3_), 2.45 and 2.56 (4H, AB system, *J* = 17.6 Hz, 2CH_2_), 6.70 (1H, d, *J* = 7.2 Hz, H-Ar), 6.80 (1H, *J* = 7.2 Hz, *J* = 7.6 Hz, H-Ar), 6.97 (1H, d, *J* = 7.6 Hz, H-Ar), 7.48 (1H, s, NH). ^13^C-NMR (CDCl_3_): δ_C_ (ppm) 16.3, 27.1, 29.0, 33.6, 41.2, 54.8, 111.9, 113.7, 118.0, 120.0, 121.9, 140.8, 163.4, 168.7, 179.0, 181.6, 195.4, 202.3. Anal. Calcd. for C_25_H_27_NO_4_: C, 74.05; H, 6.71; N, 3.45%. Found: C, 73.53; H, 6.53; N, 3.37%. HRMS (ESI): *m*/*z* [M + H]^+^ calcd for C_25_H_27_NO_4_: 405.4862; found: 405.4857.

*7-Chloro-3′,3′,6′,6′-tetramethyl-3′,4′,6′,7′-tetrahydrospiro[indoline-3,9′-xanthene]-1′,2,8′(2′H,5′H)-trione* (**3f**). White powder; m.p.: 270–271 °C; IR (KBr) cm^−1^: 3451, 2959, 1738, 1666, 1623, 1348, 1224, 1131. ^1^H-NMR (CDCl_3_): δ_H_ (ppm) 1.02 (6H, s, 2CH_3_), 1.12 (6H, s, 2CH_3_), 2.14 and 2.26 (4H, AB system, *J* = 16.0 Hz, 2CH_2_), 2.45 and 2.56 (4H, AB system, *J* = 17.6 Hz, 2CH_2_), 6.75 (1H, d, *J* = 7.2 Hz, H-Ar), 6.80 (1H, *J* = 7.2 Hz, *J* = 8.0 Hz, H-Ar), 7.14 (1H, d, *J* = 8.0 Hz, H-Ar), 7.48 (1H, s, NH). ^13^C-NMR (CDCl_3_): δ_C_ (ppm) 27.2, 28.9, 32.0, 46.7, 50.8, 113.3, 114.7, 120.5, 122.7, 128.4, 134.7, 140.1, 163.7, 177.9, 195.3. Anal. Calcd forC_24_H_24_ClNO_4_: C, 67.68; H, 5.68; N, 3.29%. Found: C, 67.53; H, 5.61; N, 3.18%. HRMS (ESI): *m*/*z* [M + H]^+^ calcd. for C_24_H_24_ClNO_4_: 425.1394; found: 425.1391.

*3′,4′,6′,7′-Tetrahydrospiro[indoline-3,9′-xanthene]-1′,2,8′(2′H,5′H)-trione* (**3g**). White powder; m.p.: 336–337 °C (m.p. 336 °C) [31]; ^1^H-NMR (CDCl_3_): δ_H_(ppm) 1.60–2.09 (4H, m, CH_2_), 2.25–2.62 (4H, m, CH_2_), 2.64 (4H, t, *J* = 6.4 Hz, CH_2_), 6.83–6.89 (3H, m, H-Ar), 7.13–7.18 (1H, m, H-Ar), 7.59 (1H, s, NH). ^13^C-NMR (CDCl_3_): δ_C_ (ppm) 20.0, 27.6, 37.2, 45.8, 109.1, 114.8, 121.9, 122.7, 128.5, 133.8, 142.2, 164.9, 178.9, 195.4. Anal. Calcd. for C_20_H_17_NO_4_: C, 71.63; H, 5.11; N, 4.18%. Found: C, 71.52; H, 5.03; N, 4.35%.

*5-Methyl-3′,4′,6′,7′-tetrahydrospiro[indoline-3,9′-xanthene]-1′,2,8′(2′H,5′H)-trione* (**3h**). White powder; m.p.: 287–288 °C; IR (KBr) cm^−1^: 3312, 2957, 1743, 1668, 1618, 1350, 1314, 1167. ^1^H-NMR (CDCl_3_): δ_H_ (ppm) 1.83–2.10 (4H, m, CH_2_), 2.23 (3H, s, CH_3_), 2.26–2.66 (4H, m, CH_2_), 2.64 (4H, t, *J* = 6.8 Hz, CH_2_), 6.67 (1H, s, H-Ar), 6.72 (1H, d, *J* = 8.0 Hz, H-Ar), 6.95 (1H, d, *J* = 7.6 Hz, H-Ar), 7.59 (1H, s, NH). ^13^C-NMR (CDCl_3_): δ_C_ (ppm) 20.0, 21.2, 27.6, 37.2, 45.9, 108.8, 114.9, 123.7, 128.9, 131.2, 133.7, 139.7, 167.9, 178.9, 195.5. Anal. Calcd. for C_21_H_19_NO_4_: C, 72.19; H, 5.48; N, 4.01%. Found: C, 71.67; H, 5.33; N, 4.15%. HRMS (ESI): *m*/*z* [M + H]^+^ calcd. for C_21_H_19_NO_4_: 349.1314; found: 349.1310.

*5-Chloro-3′,4′,6′,7′-tetrahydrospiro[indoline-3,9′-xanthene]-1′,2,8′(2′H,5′H)-trione* (**3i**). White powder; m.p.: 364–365 °C; IR (KBr) cm^−1^: 3455, 2959, 1747, 1670, 1637, 1349, 1228, 1175. ^1^H-NMR (CDCl_3_): δ_H_ (ppm) 1.97–2.10 (4H, m, CH_2_), 2.23 (3H, s, CH_3_), 2.29–2.37 (4H, m, CH_2_), 2.65 (4H, t, *J* = 6.4Hz, CH_2_), 6.77 (1H, d, *J* = 8.0 Hz, H-Ar), 6.84 (1H, d, *J* = 2.0 Hz, H-Ar), 7.13 (1H, dd, *J* = 2.0 Hz, *J* = 8.0 Hz, H-Ar), 7.60 (1H, s, NH). ^13^C-NMR (CDCl_3_): δ_C_ (ppm) 19.9, 27.5, 37.1, 46.0, 110.0, 114.4, 123.2, 127.0, 128.3, 135.3, 140.9, 165.3, 178.4, 195.4. *Anal.* Calcd for C_20_H_16_ClNO_4_: C, 64.96; H, 4.36; N, 3.79%. Found: C, 64.87; H, 4.28; N, 3.61%. HRMS (ESI): *m*/*z* [M + H]^+^ calcd. for C_20_H_16_ClNO_4_: 369.0768; found: 369.0764.

*5-Fuloro-3′,3′,6′,6′-tetramethyl-3′,4′,6′,7′-tetrahydrospiro[indoline-3,9′-xanthene]-1′,2,8′(2′H,′H)-trione* (**3j**). White powder; m.p.: 302–303 °C; IR (KBr) cm^−1^: 3457, 2958, 1732, 1667, 1618, 1492, 1349, 1222, 1167. ^1^H-NMR (CDCl_3_): δ_H_ (ppm) 1.04 (6H, s, 2CH_3_), 1.12 (6H, s, 2CH_3_), 2.15 and 2.27 (4H, AB system, *J* = 16.0 Hz, 2CH_2_), 2.45 and 2.57 (4H, AB system, *J* = 17.6 Hz, 2CH_2_), 6.60 (1H, dd, *J* = 2.0 Hz, *J* = 7.6 Hz, H-Ar), 6.72 (1H, *J* = 4.4 Hz, *J* = 8.4 Hz, H-Ar), 6.80–6.85 (1H, m, H-Ar), 8.16 (1H, s, NH). ^13^C-NMR (CDCl_3_): δ_C_ (ppm) 27.3, 28.9, 32.0, 41.2, 46.2, 50.9, 110.1, 113.3, 114.6, 134.9, 138.6, 157.7, 160.1, 163.8, 178.7, 195.4. Anal. Calcd. for C_24_H_24_FNO_4_: C, 70.40; H, 5.91; N, 3.42%. Found: C, 69.93; H, 5.86; N, 3.35%. HRMS (ESI): *m*/*z* [M + H]^+^ calcd. for C_24_H_24_FNO_4_: 409.1689; found: 409.1685.

*5-Bromo-3′,4′,6′,7′-tetrahydrospiro[indoline-3,9′-xanthene]-1′,2,8′(2′H,5′H)-trione* (**3k**). White powder; m.p.: 288–289 °C; IR (KBr) cm^−1^: 3447, 2959, 1733, 1668, 1619, 1495, 1349, 1224, 1169. ^1^H-NMR (CDCl_3_): δ_H_ (ppm) 1.98–2.10 (4H, m, 2CH_2_), 2.29–2.37 (4H, m, 2CH_2_), 2.65 (4H, t, *J* = 6.4 Hz, 2CH_2_), 6.72 (1H, d, *J* = 8.4 Hz, H-Ar), 6.97 (1H, d, *J* = 1.6 Hz, H-Ar), 7.25–7.28 (1H, m, H-Ar), 7.71 (1H, s, NH). ^13^C-NMR (CDCl_3_): δ_C_ (ppm) 19.9, 27.5, 37.1, 45.9, 110.5, 114.3, 114.4, 125.9, 131.2, 135.7, 141.4, 165.3, 178.3, 195.4. Anal. Calcd. for C_20_H_16_BrNO_4_: C, 57.99; H, 3.89; N, 3.38%. Found: C, 57.71; H, 3.81; N, 3.42%. HRMS (ESI): *m*/*z* [M + H]^+^ calcd. for C_20_H_16_BrNO_4_: 413.0263; found: 413.0259.

*5-Bromo-3′,3′,6′,6′-tetramethyl-3′,4′,6′,7′-tetrahydrospiro[indoline-3,9′-xanthene]-1′,2,8′(2′H,5′H)-trione* (**3l**). White powder; m.p.: 290 °C (290 °C) [15]; IR (KBr) cm^−1^: 3449, 2958, 1731, 1665, 1617, 1347, 1223, 1165. ^1^H-NMR (CDCl_3_): δ_H_ (ppm) 1.05 (6H, s, 2CH_3_), 1.11 (6H, s, 2CH_3_), 2.15 and 2.25 (4H, AB system, *J* = 16.4 Hz, 2CH_2_), 2.47 and 2.56 (4H, AB system, *J* = 17.6 Hz, 2CH_2_), 6.72 (1H, d, *J* = 8.4 Hz, H-Ar), 6.95 (1H, d, *J* = 2.0 Hz, H-Ar), 7.26(1H, m, H-Ar), 7.98 (1H, s, NH). ^13^C-NMR (CDCl_3_): δ_C_ (ppm) 27.5, 28.7, 32.0, 41.2, 45.8, 50.9, 110.9, 113.2, 114.3, 125.5, 131.3, 135.6, 141.5, 164.0, 178.4, 195.6. Anal. Calcd. for C_24_H_24_BrNO_4_: C, 61.28; H, 5.14%; N, 2.98%. Found: C, 61.03; H, 5.17; N, 2.92%. 

*6-Fuloro-3′,3′,6′,6′-tetramethyl-3′,4′,6′,7′-tetrahydrospiro[indoline-3,9′-xanthene]-1′,2,8′(2′H,5′H)-trione* (**3m**). White powder; m.p.: 281–283 °C; IR (KBr) cm^−1^: 3453, 2959, 1739, 1664, 1619, 1347, 1223, 1130. ^1^H-NMR (CDCl_3_): δ_H_ (ppm) 1.04 (6H, s, 2CH_3_), 1.13 (6H, s, 2CH_3_), 2.15 and 2.28 (4H, AB system, *J* = 16.4 Hz, 2CH_2_), 2.46 and 2.58 (4H, AB system, *J* = 17.6 Hz, 2CH_2_), 6.74 (1H, d, *J* = 6.8 Hz, H-Ar), 6.70–7.03 (1H, m, H-Ar), 8.03 (1H, s, NH). ^13^C-NMR (CDCl_3_): δ_C_ (ppm) 27.2, 28.9, 32.0, 41.2, 45.4, 50.9, 112.9, 113.2, 121.9, 123.4, 124.8, 132.6, 143.6, 163.8, 178.6, 195.5. Anal. Calcd. for C_24_H_24_FNO_4_: C, 70.40; H, 5.91; N, 3.42%. Found: C, 69.99; H, 5.85; N, 3.47%. HRMS (ESI): *m*/*z* [M + H]^+^ calcd for C_24_H_24_FNO_4_: 409.1689; found: 409.1685.

## 4. Conclusions

The present report describes the synthesis of spiro[indoline-3,9′-xanthene]trione derivatives in good yields, catalyzed by Mg(ClO_4_)_2_ at 80 °C in aqueous ethanol media. This protocol is efficient, simple, mild, eco-friendly, and also advantageous in terms of short reaction time and easy workup procedure.

## Figures and Tables

**Table 1:** Solvent effects on the reaction of isatin and dimedone, in the presence of 10 mol % magnesium perchlorate ^a^.

Entry	Solvent/T (°C)	Time (h)	Yield ^b^ (%)
1	PhCH_3_(reflux)	24	Trace
2	CH_2_Cl_2_(reflux)	24	Trace
3	CH_3_OH(reflux)	12	64
4	C_2_H_5_OH(reflux)	12	71
5	C_2_H_5_OH/H_2_O(1/1)(80)	10	83
6	H_2_O(80)	12	77
7	CH_3_CN(reflux)	12	60
8	THF(reflux)	12	58

^a^ Isatin (2 mmol), dimedone (4 mmol); solvent (5 mL), Mg(ClO_4_)_2_ (10%mol). ^b^ Isolated yield.

**Table 2:** Synthesis of spiro[indoline-3,9′-xanthene]trione **3**.

Product	R_1_	R_2_	Time (h)	Yield ^a^ (%)
**3a**	H	CH_3_	10	83
**3b**	5-Me	CH_3_	12	75
**3c**	5-Cl	CH_3_	10	79
**3d**	5-NO_2_	CH_3_	10	81
**3e**	7-Me	CH_3_	12	71
**3f**	7-Cl	CH_3_	10	78
**3g**	H	H	10	80
**3h**	5-Me	H	12	69
**3i**	5-Cl	H	10	82
**3j**	5-F	CH_3_	10	83
**3k**	5-Br	H	10	71
**3l**	5-Br	CH_3_	10	78
**3m**	6-F	CH_3_	10	85

^a^ Isolated yield.

## Supplementary Materials

Supplementary materials are available online.
