# Supplementary material for: An Efficient Synthesis of Spiro[indoline-3,9′-xanthene]trione Derivatives Catalyzed by Magnesium Perchlorate
Source: Molecules. 2017 Aug 4;22(8):1295. doi: 10.3390/molecules22081295 (PMC6152258; doi:10.3390/molecules22081295)

An Efficient Synthesis of Spiro[indoline-3,9′-xanthene]trione Derivatives Catalyzed by Magnesium Perchlorate

Chunfeng Chen[†‡], Chunlei Lv[‡], Jianfeng Liang[‡], Jianqing Jin[‡], Runpu Shen[†], Chunlei Wu[†]*

[†]. Department of Chemistry and Chemical Engineering, Shaoxing University, Shaoxing, Zhejiang 312000, P. R. of China

[‡]. Zhejiang Medicine Co., Ltd., Shaoxing, Zhejiang, 312000, P. R. of China

Email: wuchunlei2006@usx.edu.cn

Received Date; Accepted Date

Abstract *:* A simple and efficient method for the synthesis of spiro[indoline-3,9'-xanthene]trione derivatives by means of condensation between isatins and 1,3-cyclohexanedione in the presence of catalytic amount of magnesium perchlorate at 80 ℃ in 50% aqueous ethanol medium has been described. Notably, the present method offers desirable advantages of good yields, simplicity of work-up procedure, easy purification, reduced reaction times.

Keywords **:** Aqueous; isatin; spiro[indoline-3,9′-xanthene]trione; magnesium perchlorate

The ^{1}H NMR and ^{13}C NMR spectrum of the product 3a-3m:

3′,3′,6′,6′-Tetramethyl-3′,4′,6′,7′-tetrahydrospiro[indoline-3,9′-xanthene]-1′,2,8′(2′*H*, 5′*H*)-trione (3a)

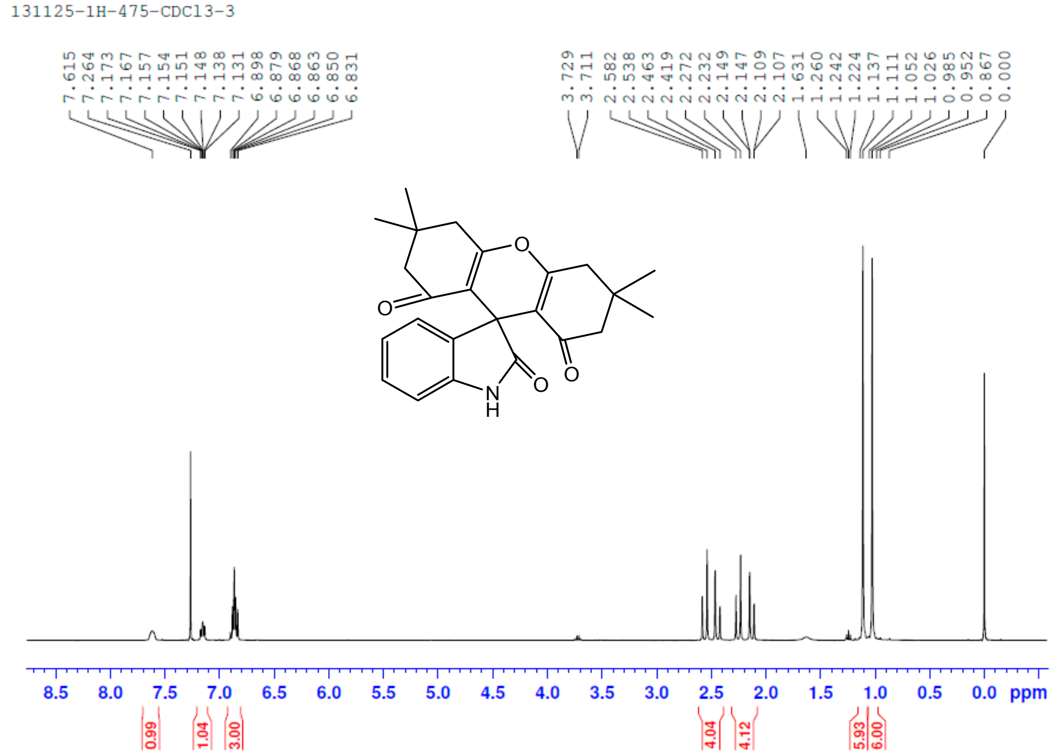

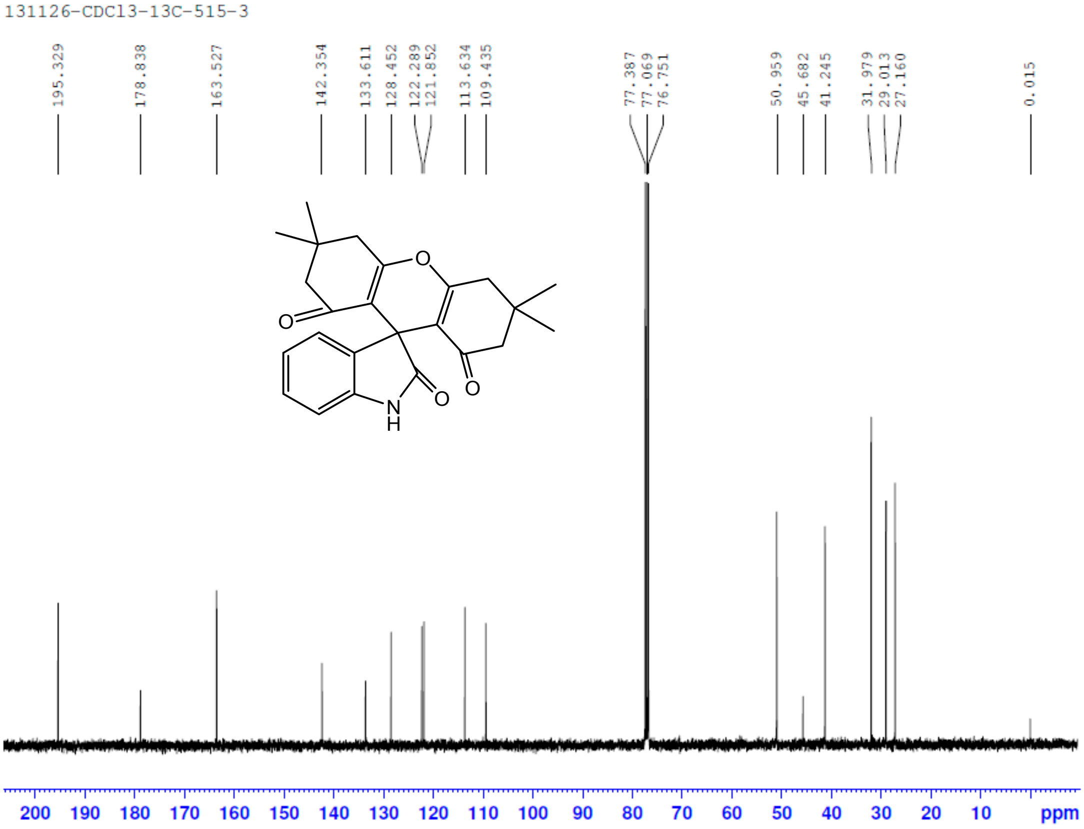

5,3′,3′,6′,6′-Pentamethyl-3′,4′,6′,7′-tetrahydrospiro[indoline-3,9′-xanthene]- 1′,2,8′ (2′*H*, 5′*H*)-trione(3b)

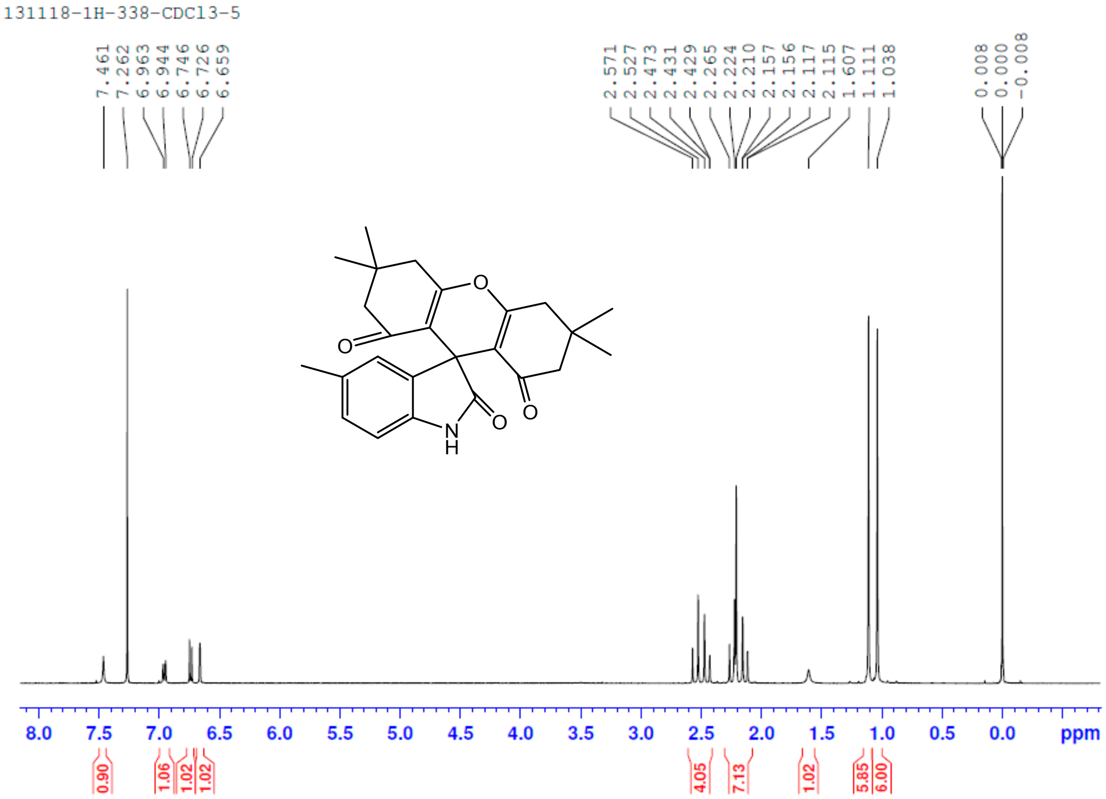

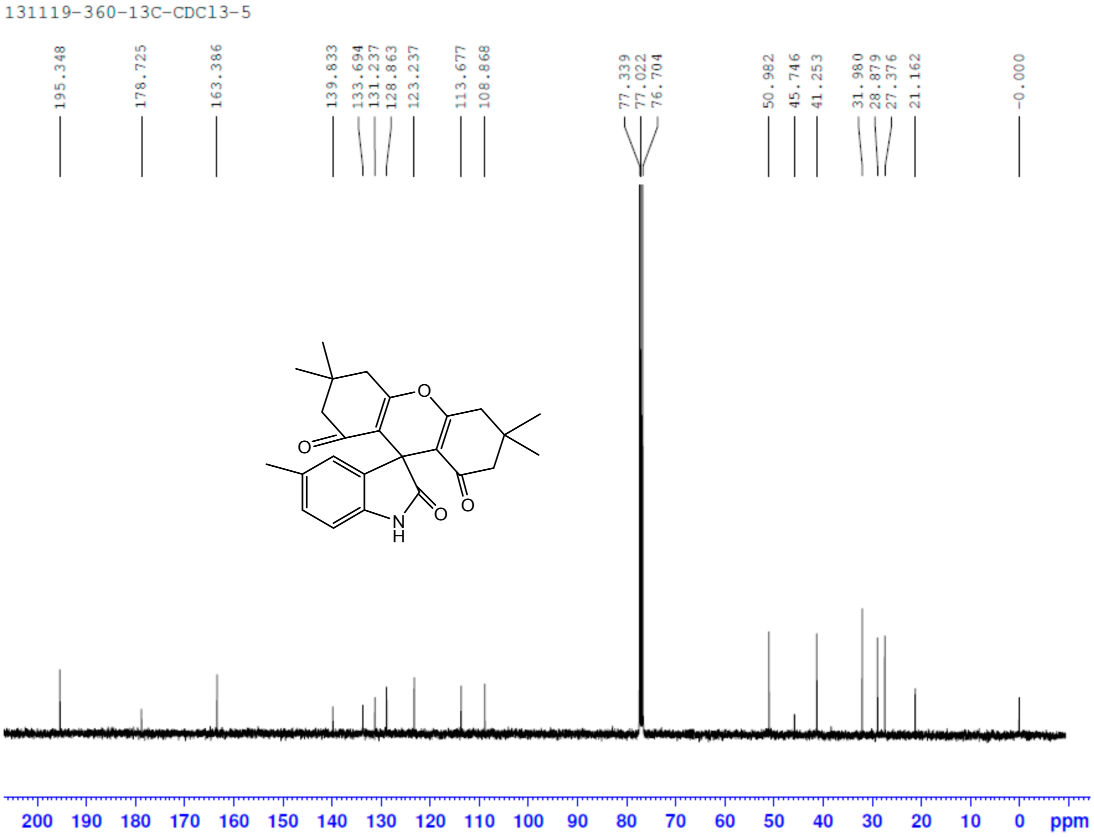

5-Chloro-3′,3′,6′,6′-tetramethyl-3′,4′,6′,7′-tetrahydrospiro[indoline-3,9′- xanthene]-1′,2,8′(2′*H*, 5′*H*)-trione (3c)

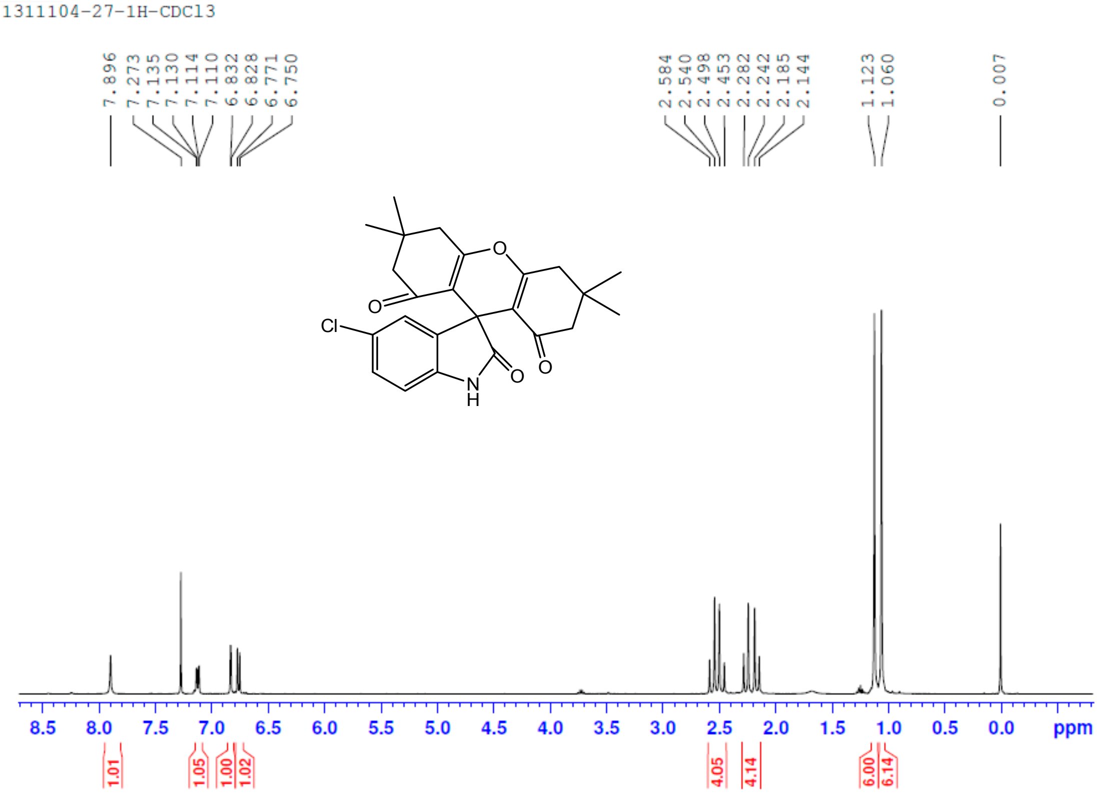

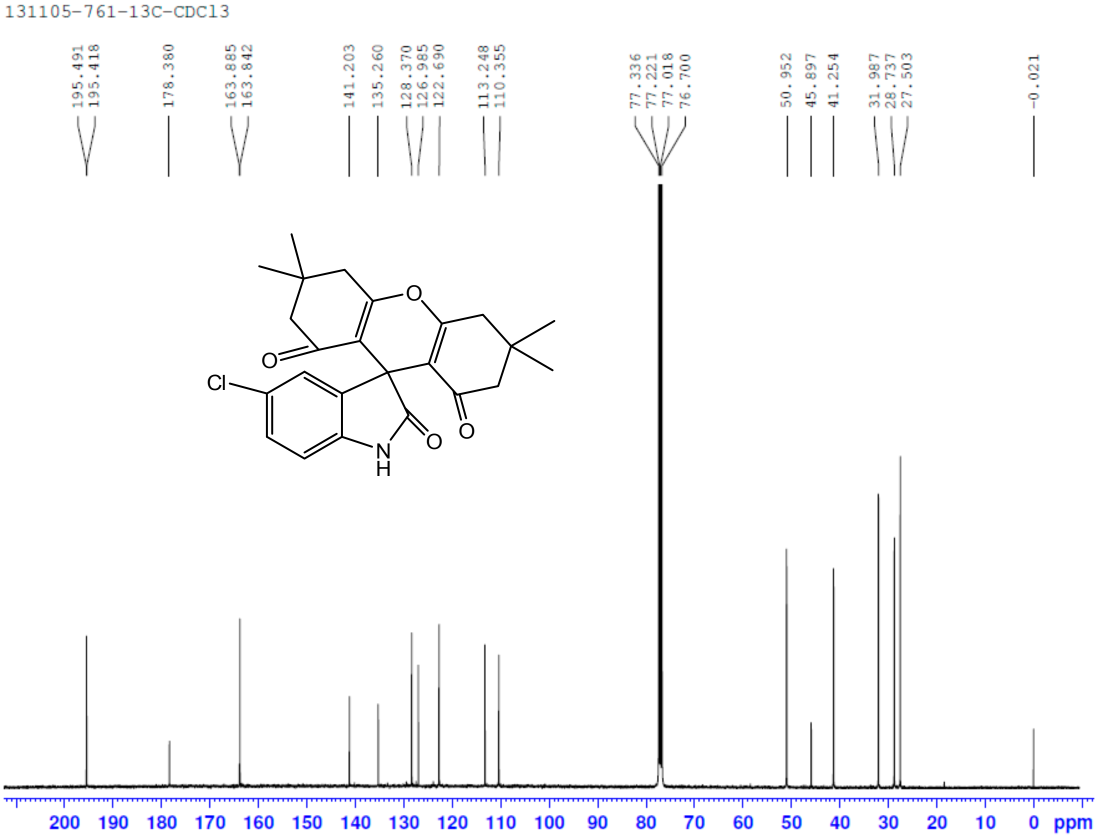

5-Nitro-3′,3′,6′,6′-tetramethyl-3′,4′,6′,7′-tetrahydrospiro[indoline-3,9′-xanthene]-1′,2,8′(2′*H*, 5′*H*)-trione (3d)

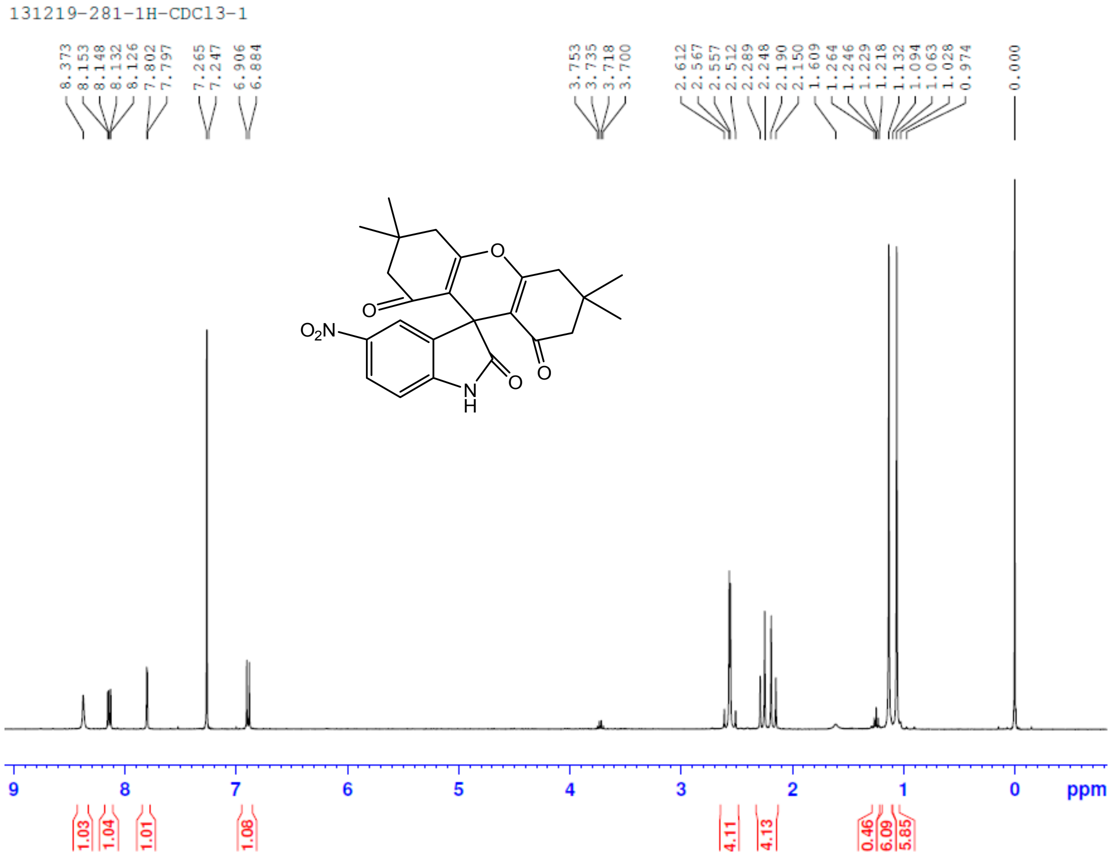

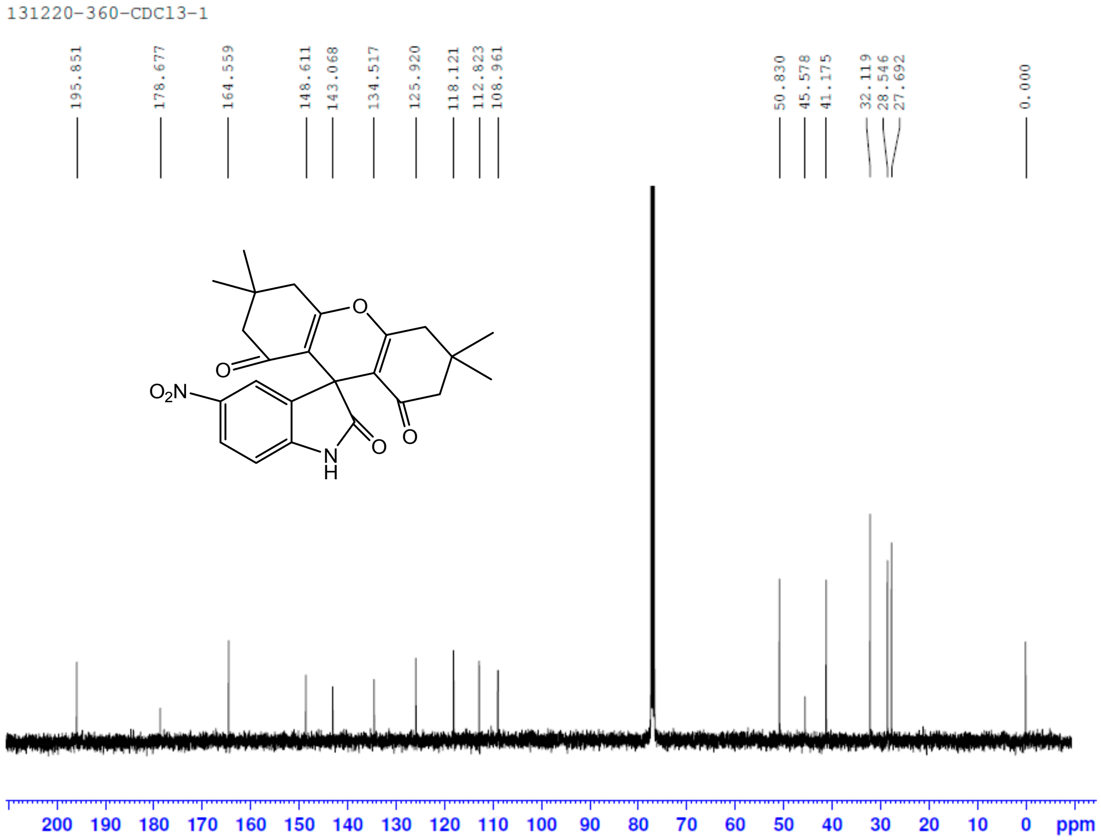

7,3′,3′,6′,6′-Pentamethyl-3′,4′,6′,7′-tetrahydrospiro[indoline-3,9′-xanthene]-1′,2,8′(2′*H*, 5′*H*)-trione (3e).

131118-1H-339-CDCl3-6

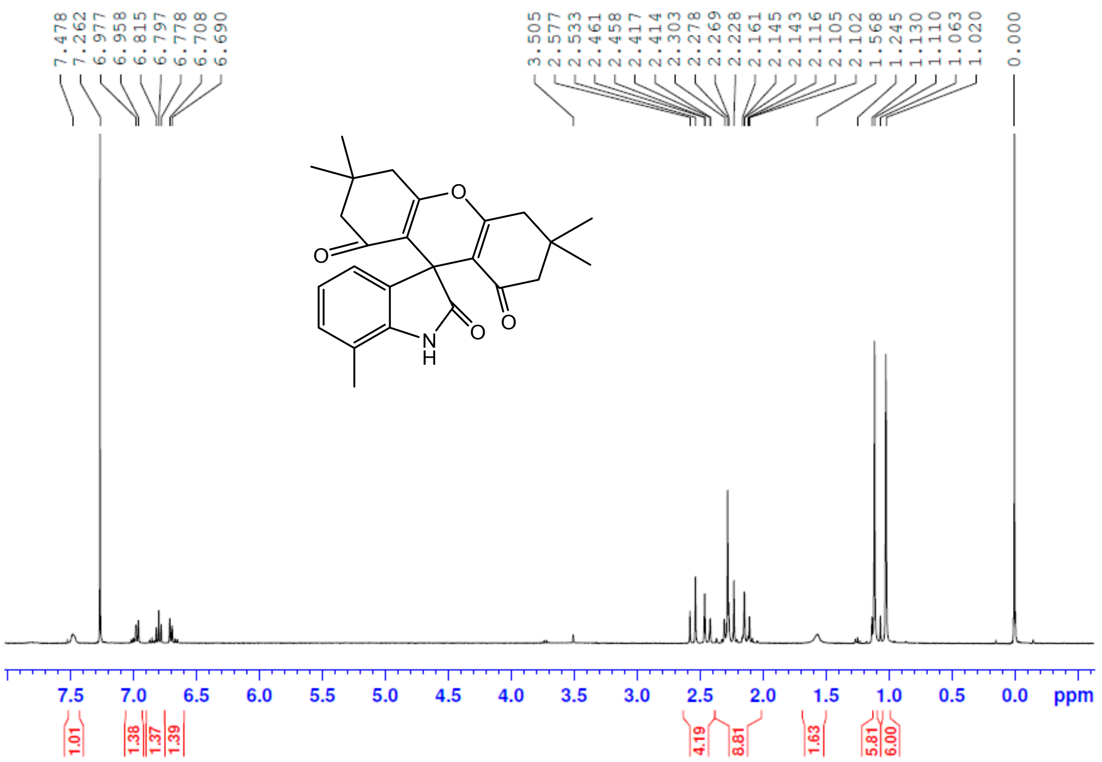

131119-361-13C-CDCl3-6

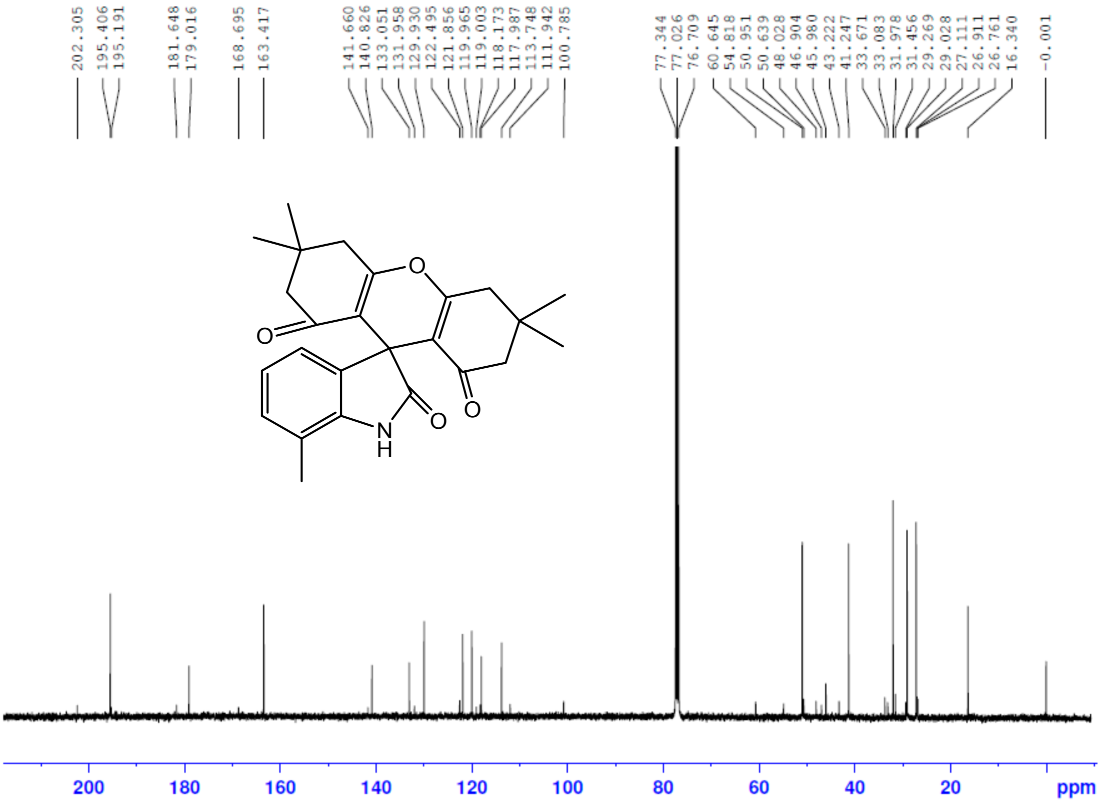

7-Chloro-3′,3′,6′,6′-tetramethyl-3′,4′,6′,7′-tetrahydrospiro[indoline-3,9′- xanthene]-1′,2,8′(2′*H*, 5′*H*)-trione (3f)

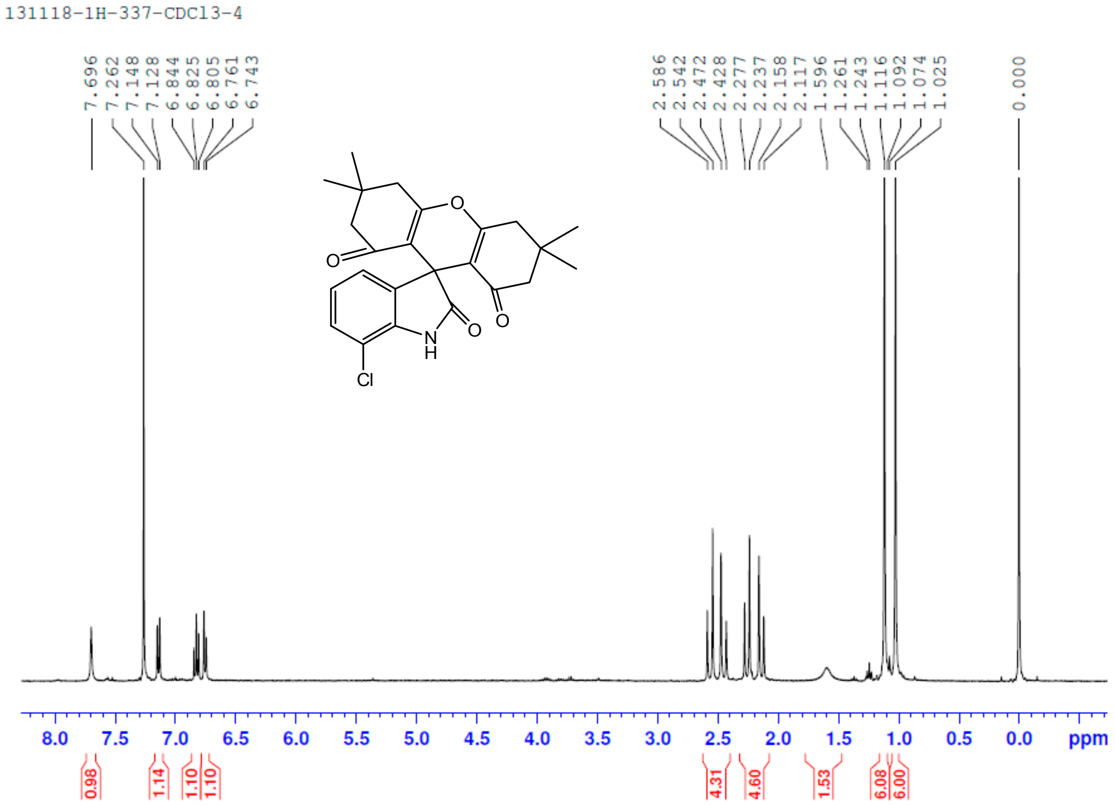

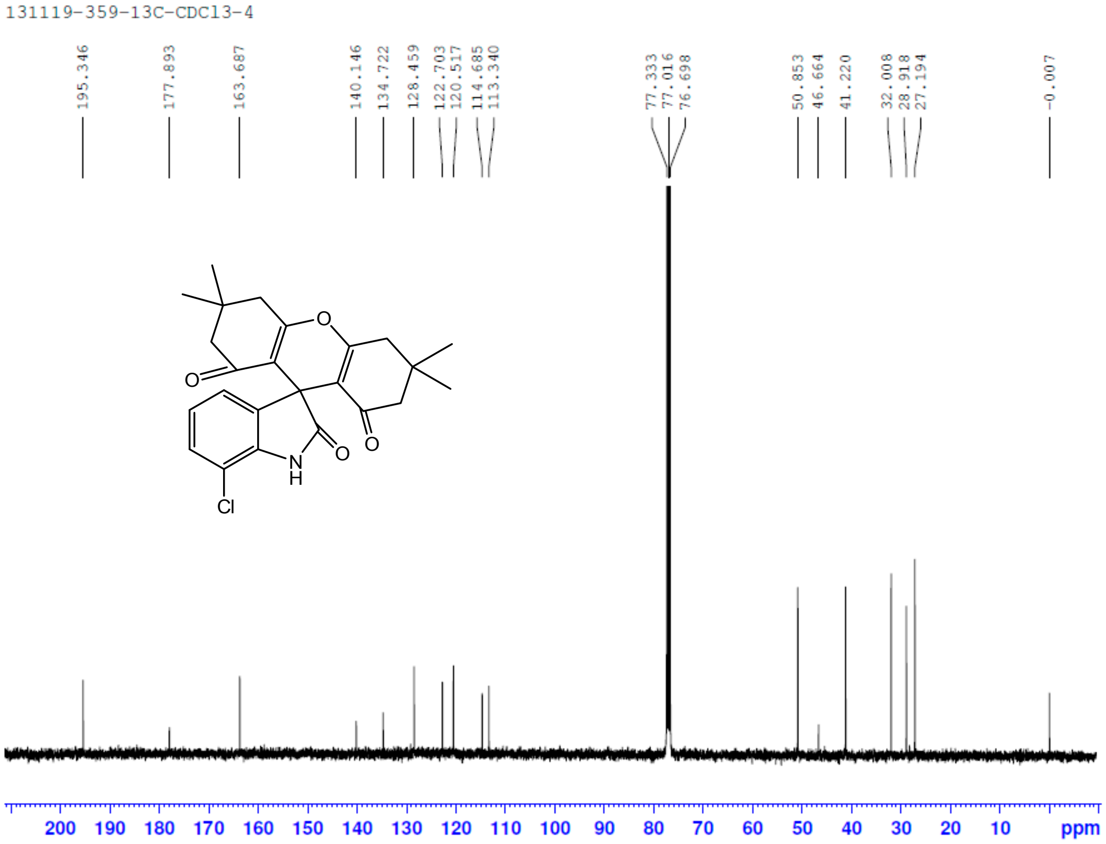

3′,4′,6′,7′-Tetrahydrospiro[indoline-3,9′-xanthene]-1′,2,8′(2′*H*, 5′*H*)-trione (3g)

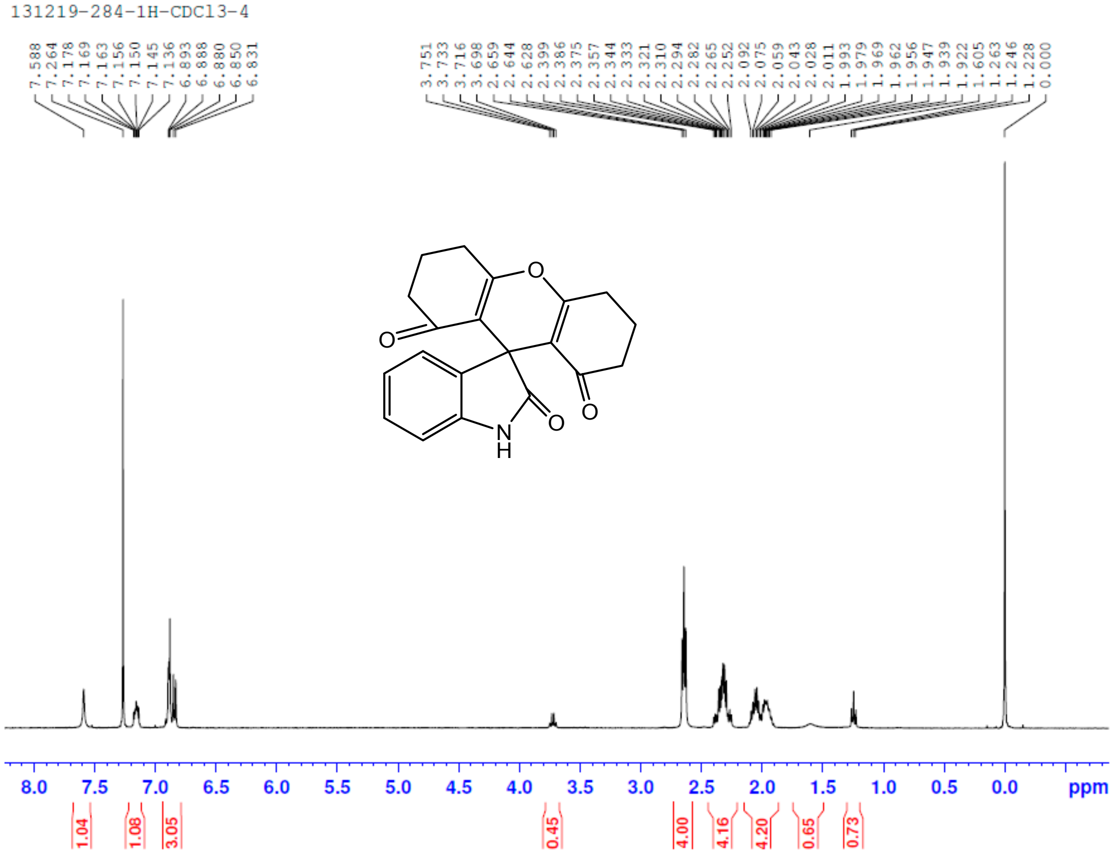

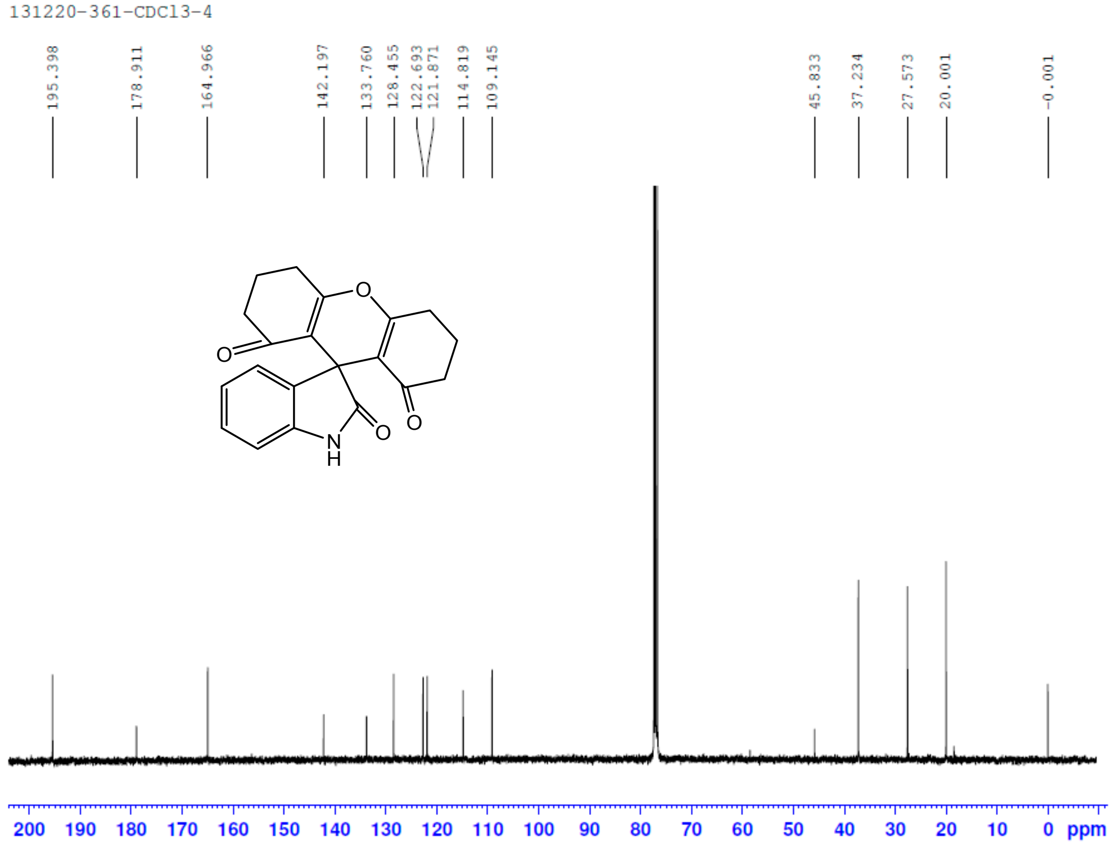

5-Methyl-3′,4′,6′,7′-tetrahydrospiro[indoline-3,9′-xanthene]-1′,2,8′(2′*H*, 5′*H*)-trione (3h)

140425-451-HNMR-yangzaen

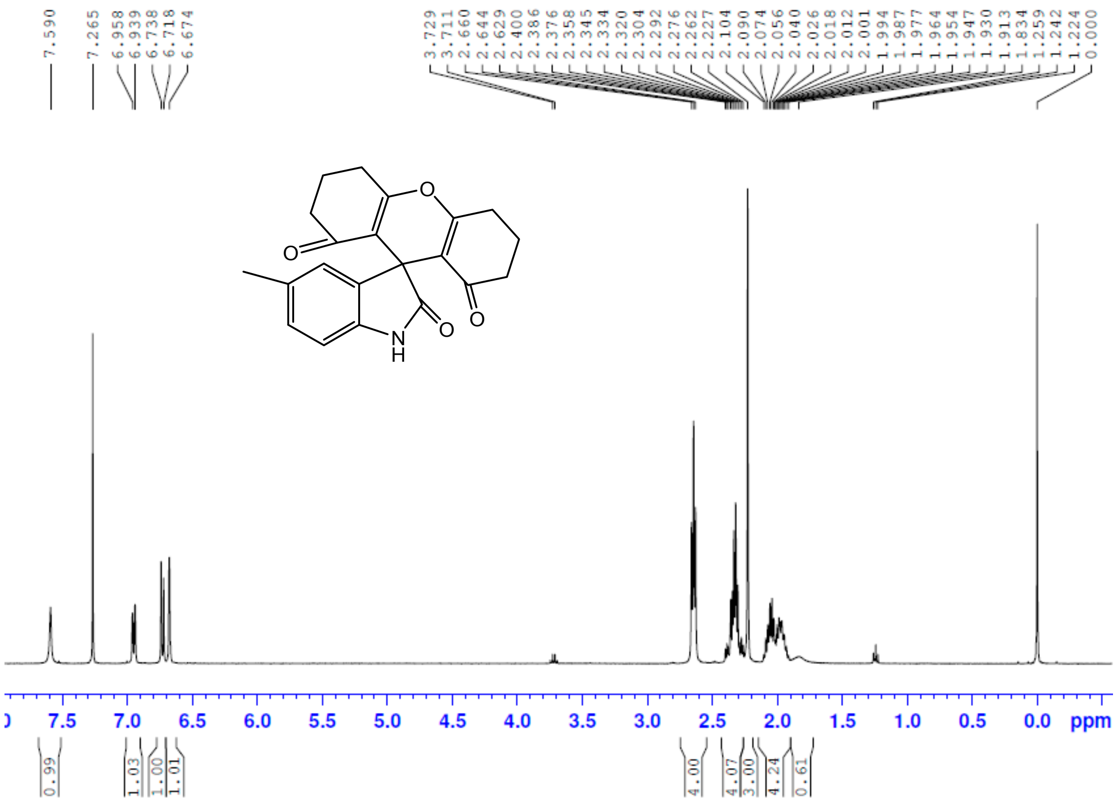

140428-506-CDCl3-yangzaen

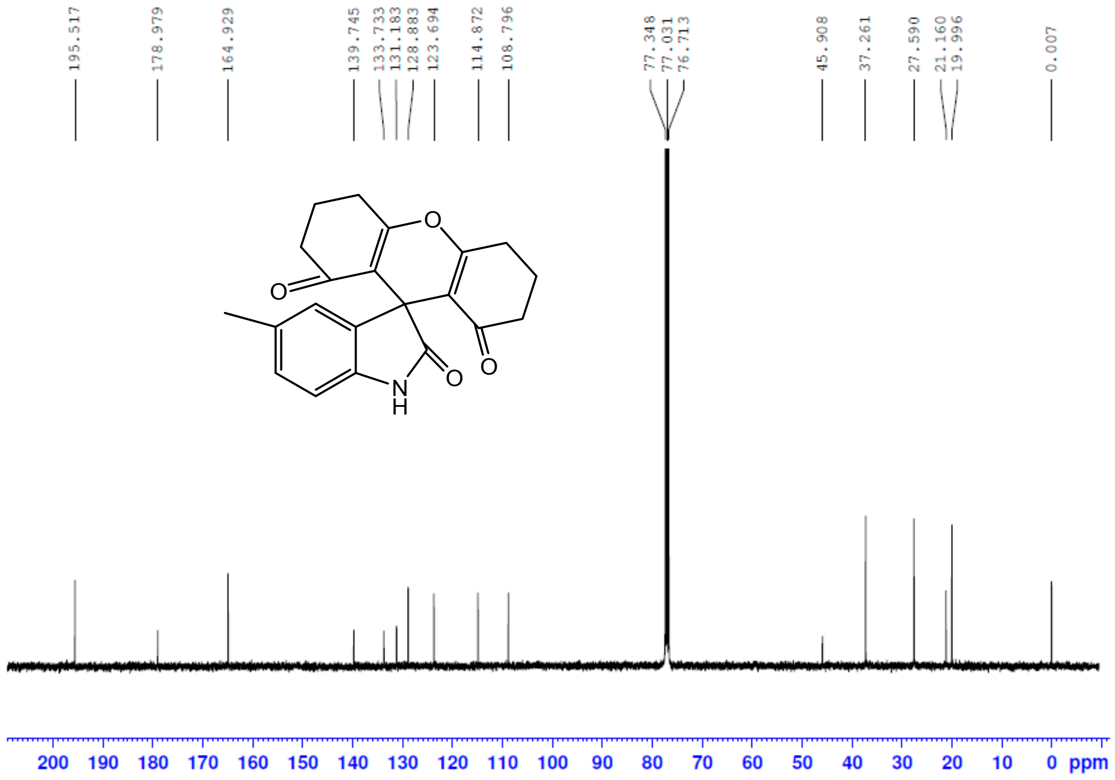

5-Chloro-3′,4′,6′,7′-tetrahydrospiro[indoline-3,9′-xanthene]-1′,2,8′(2′*H*, 5′*H*)-trione (3i)

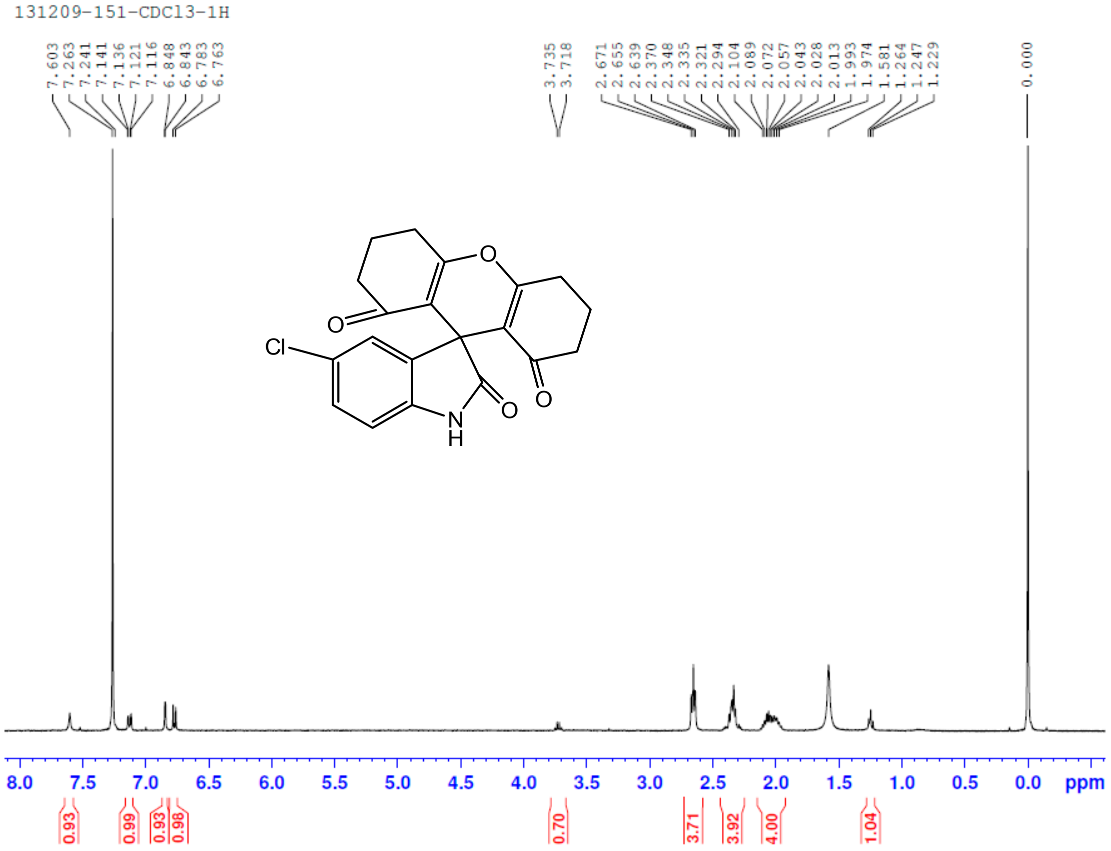

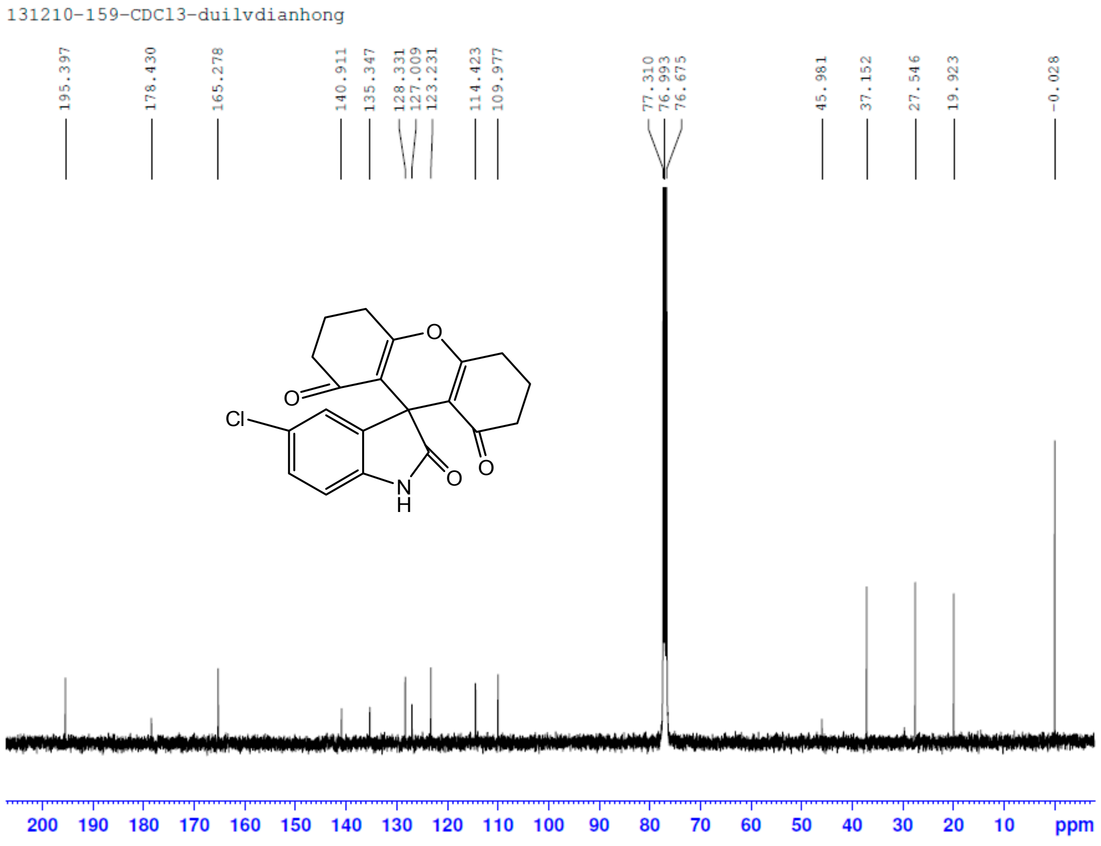

5-Fuloro-3′,3′,6′,6′-tetramethyl-3′,4′,6′,7′-tetrahydrospiro[indoline-3,9′- xanthene]-1′,2,8′(2′*H*, 5′*H*)-trione (3j)

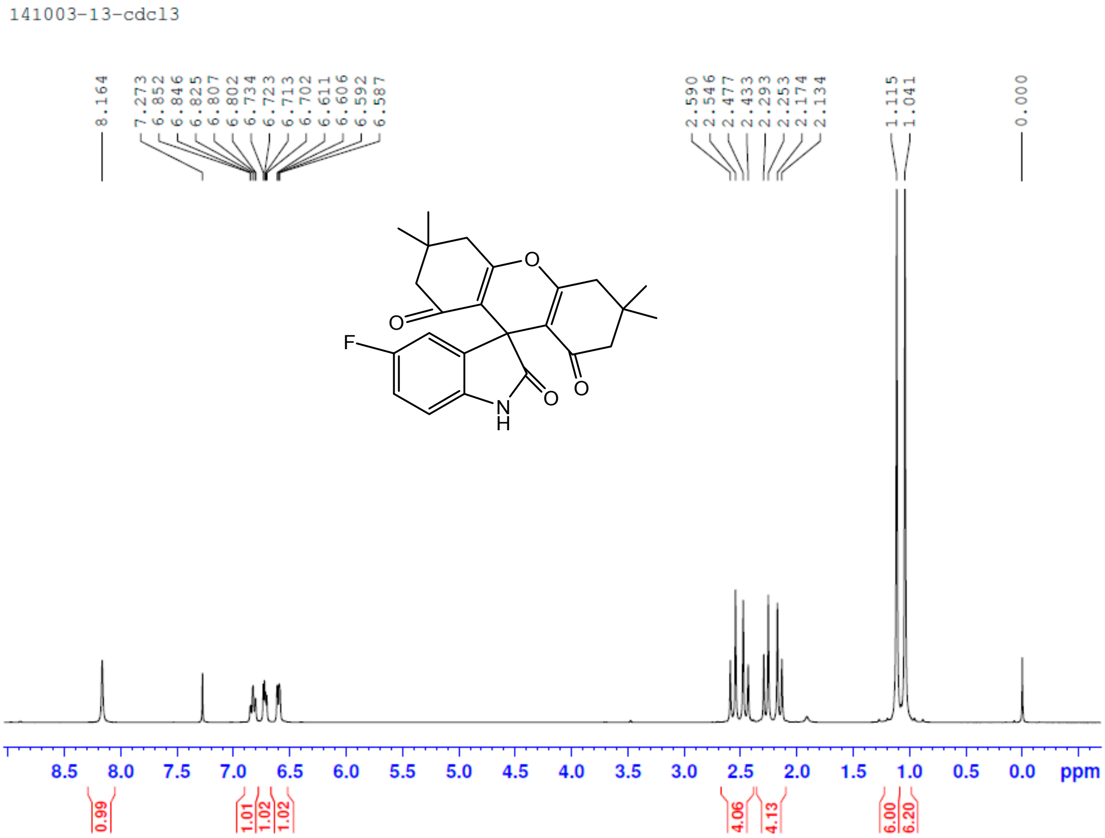

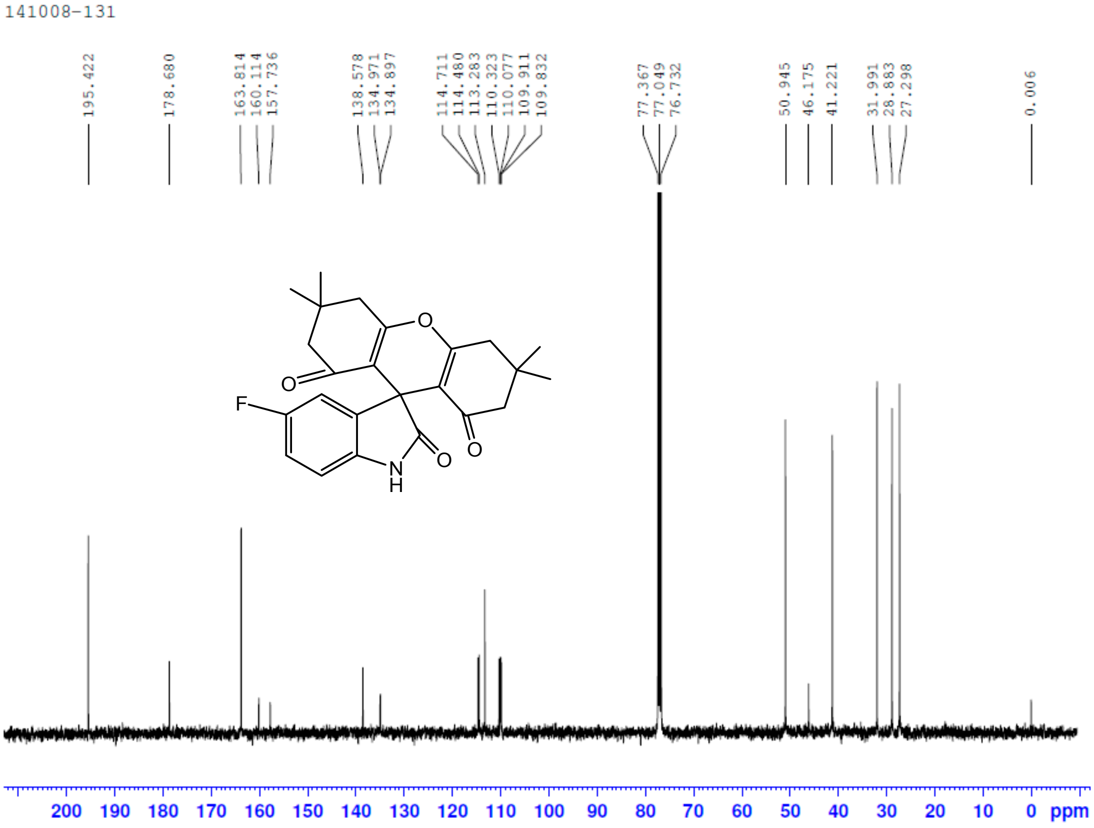

5-Bromo-3′,4′,6′,7′-tetrahydrospiro[indoline-3,9′-xanthene]-1′,2,8′(2′*H*, 5′*H*)-trione (3k)

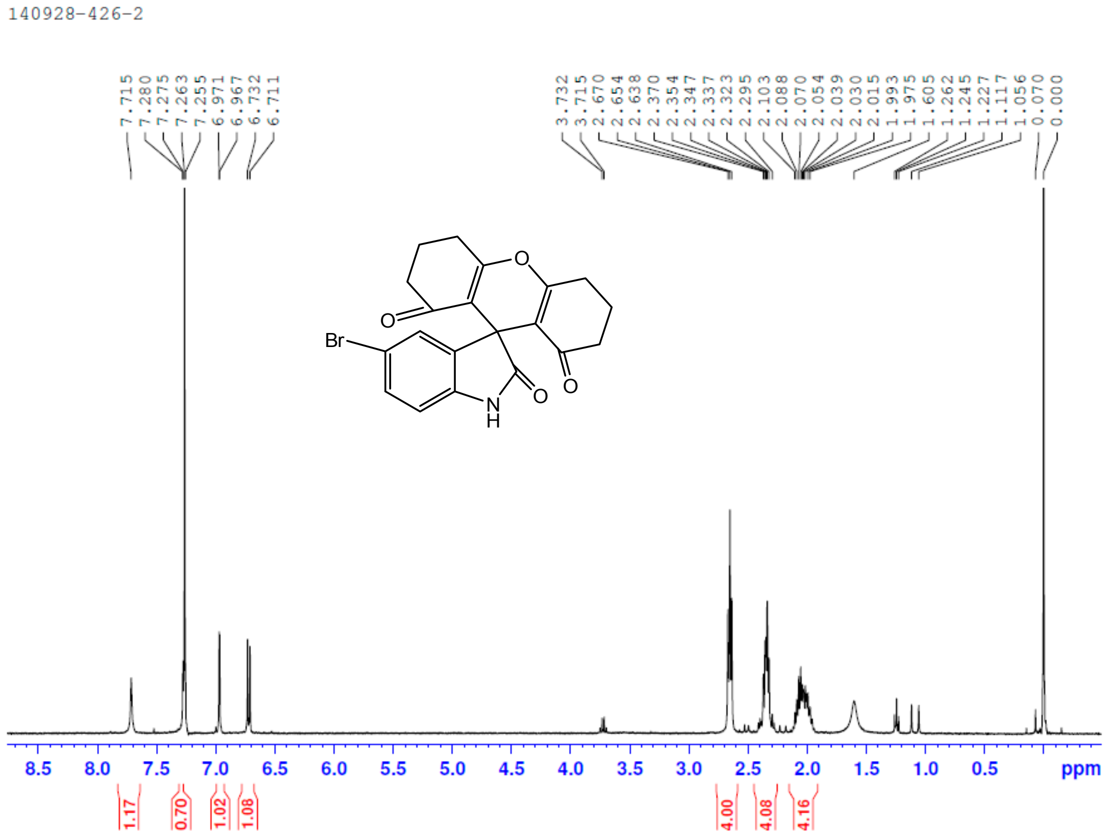

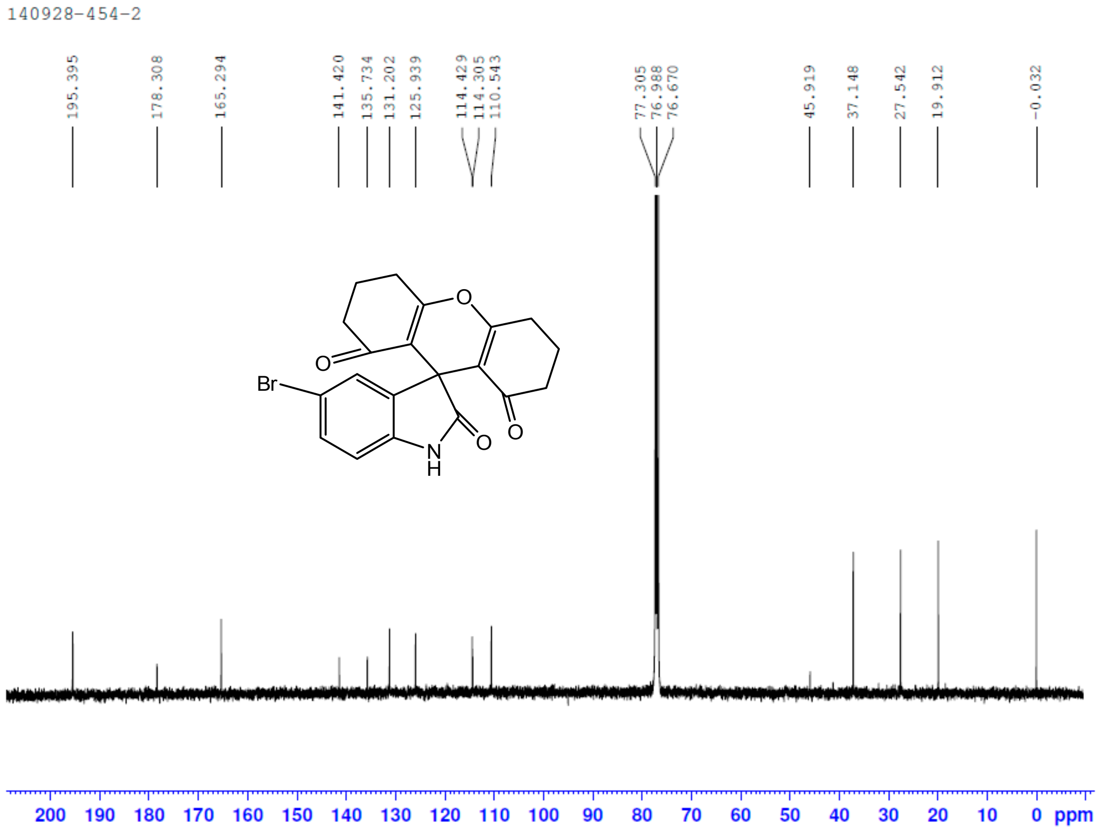

5-Bromo-3′,3′,6′,6′-tetramethyl-3′,4′,6′,7′-tetrahydrospiro[indoline-3,9′- xanthene]-1′,2,8′(2′*H*, 5′*H*)-trione (3l)

140928-425-1

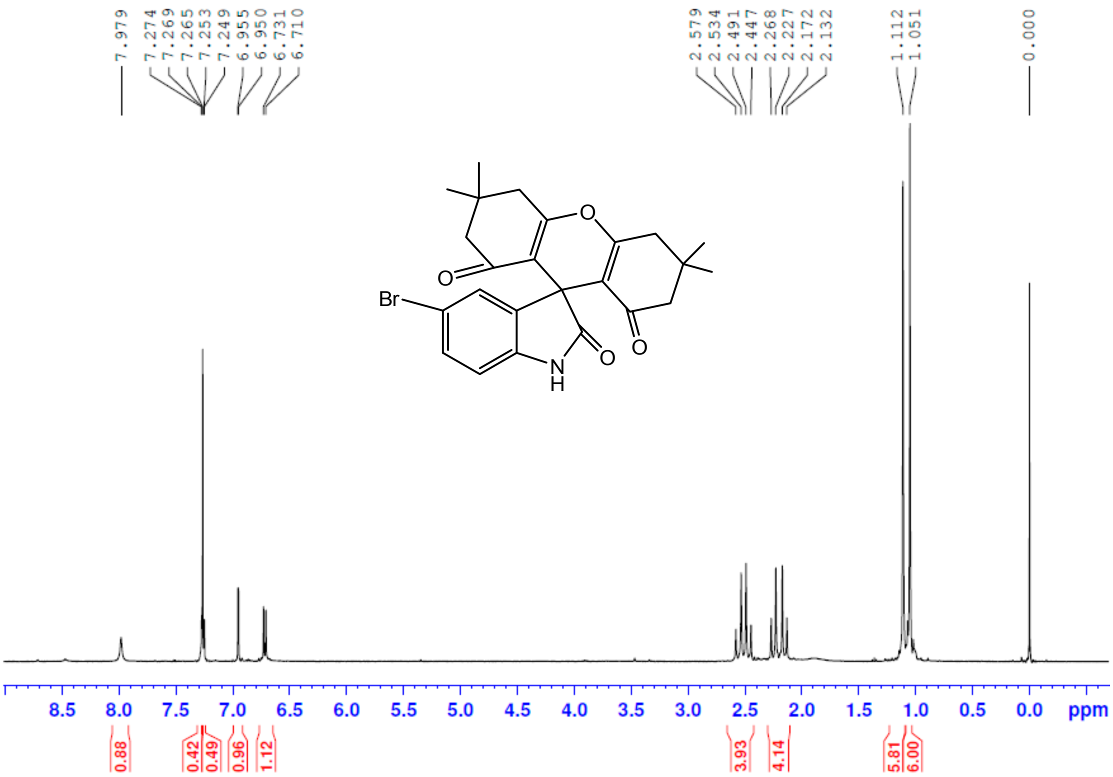

140928-453-1

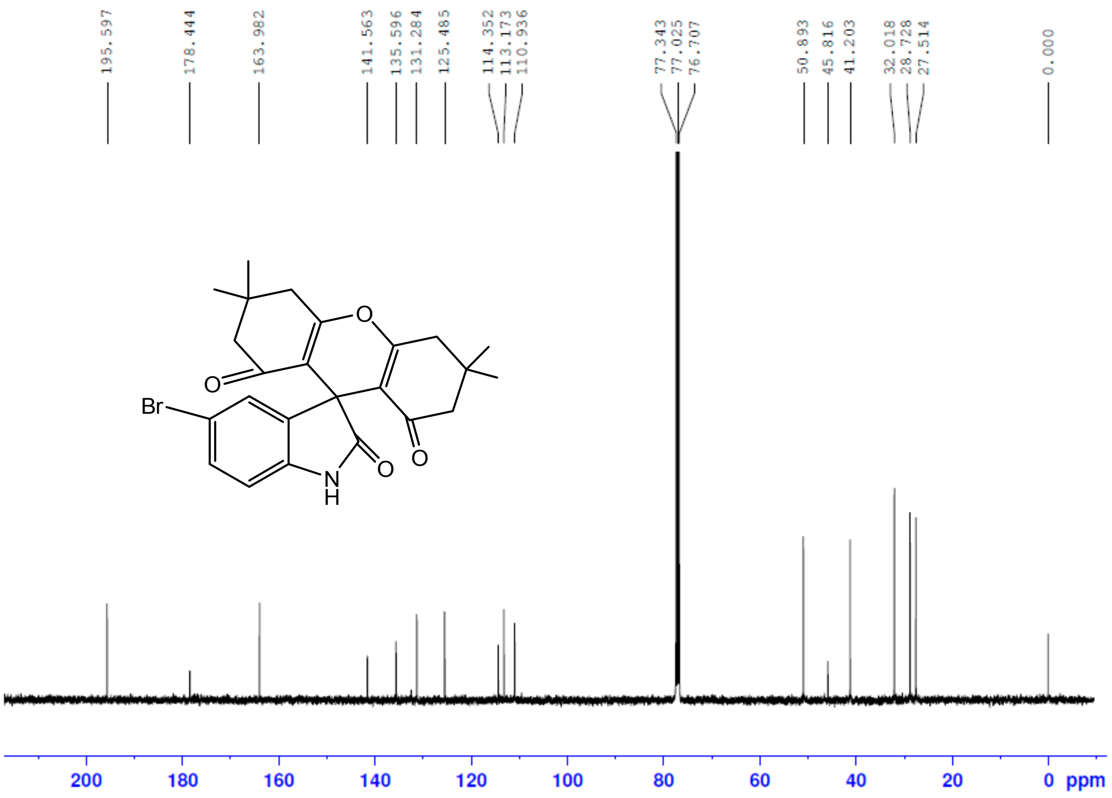

6-Fuloro-3′,3′,6′,6′-tetramethyl-3′,4′,6′,7′-tetrahydrospiro[indoline-3,9′-xanthene]-1′,2,8′(2′*H*, 5′*H*)-trione (3m).

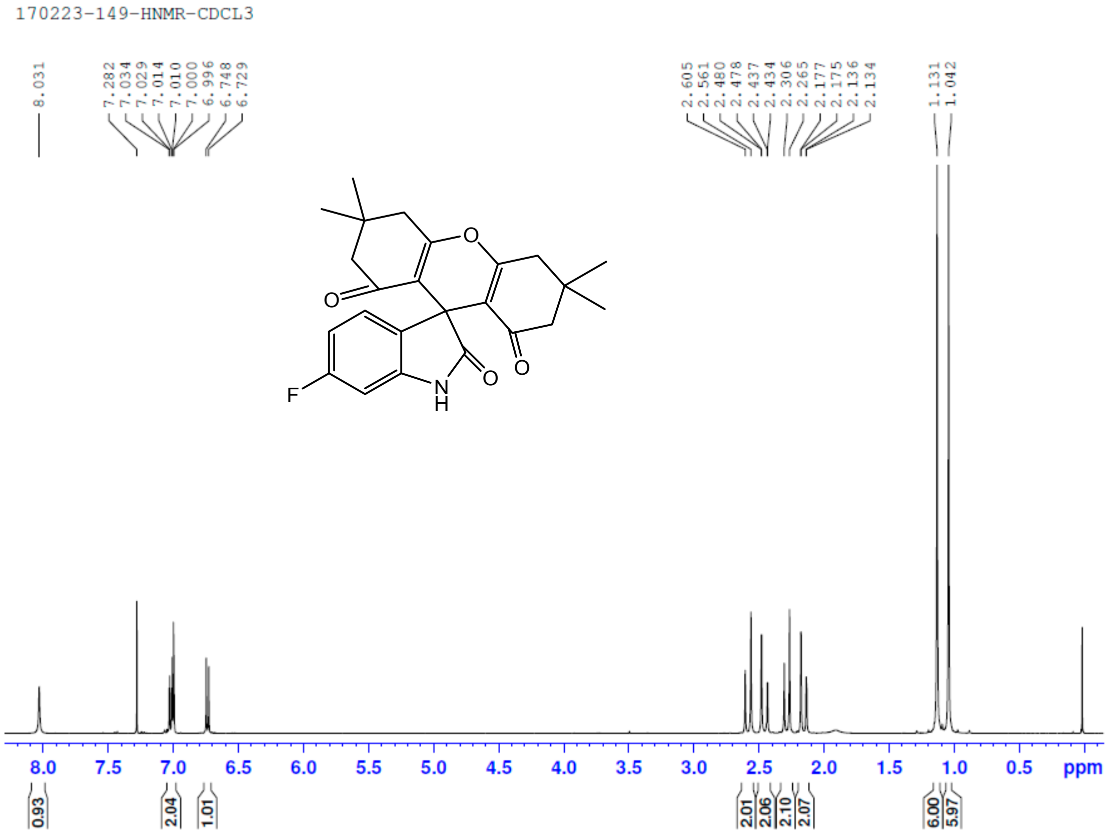

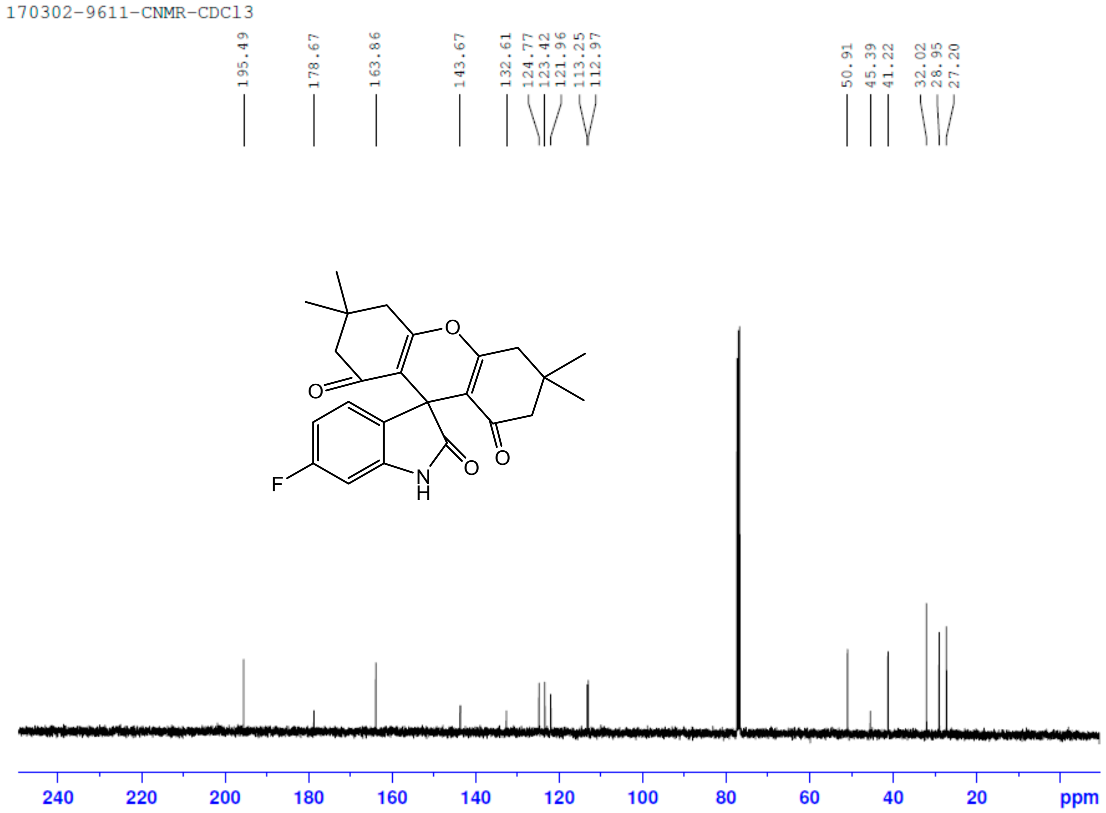

Supplement: Supplementary file 1 [file molecules-22-01295-s001.pdf]
